# Like Mother Like Child: Do Fearful Sows Have Fearful Piglets?

**DOI:** 10.3390/ani11051232

**Published:** 2021-04-24

**Authors:** Hazel B. Rooney, Oceane Schmitt, Alexandra Courty, Peadar G. Lawlor, Keelin O’Driscoll

**Affiliations:** 1 Pig Development Department, Teagasc Animal and Grassland Research and Innovation Centre, Moorepark, P61 C996 Fermoy, Co., Cork, Ireland; hazel.rooney@alltech.com (H.B.R.); Oceane.Schmitt@vetmeduni.ac.at (O.S.); courty.alexandra@gmail.com (A.C.); peadar.lawlor@teagasc.ie (P.G.L.); 2 Department of Animal Production, Easter Bush Veterinary Centre, Royal (Dick) School of Veterinary Studies, The University of Edinburgh, Easter Bush Campus, Midlothian EH25 9RG, UK; 3 Animal Behaviour and Welfare Team, Animal and Veterinary Sciences Research Group, SRUC, West Mains Road, Edinburgh EH9 3JG, UK

**Keywords:** back test, coping style, cortisol, human fear, human approach test, novel environment test, nursing behaviour, piglets, prenatal stress, sows

## Abstract

**Simple Summary:**

Early life and gestational experience influence the behavioural development of the offspring. This study investigated the relationship between gestating gilts’ fear of humans and cortisol levels and their feeding and maternal behaviour, and the personality (coping style, human fear) and growth of their piglets. Gilts were classified as fearful or friendly after four human approach tests performed between d 104 and d 111 of gestation, cortisol level was assessed between d 90 and d 108 of gestation, and maternal behaviour evaluated at d 13 of lactation. Piglets were submitted to a back test at 13 days old, and to a human approach test and an open field test at 20 days old. Fearful gilts had higher cortisol levels than friendly gilts. Piglets from friendly gilts tended to have a more active response to the back test, less freezing reaction in the open field test, and accepted human contact more than piglets from fearful gilts. The results of this study support the hypothesis that the fearfulness of gilts towards humans is related to their stress levels, and that both could influence the behavioural profile of their offspring.

**Abstract:**

Gestational and early life experiences affect subsequent behavioural and physical development. The objective of the current study was to investigate associations between gilts’ fear of humans, gestational stress level, and feeding and maternal behaviour, as well as how these related to aspects of the personality and growth of their offspring. A total of 37 gilts were used. Four human approach tests were performed between d 104 and d 111 of gestation to classify gilts as fearful or friendly. Gilt feeding behaviour and salivary cortisol concentration was measured between d 90 and d 108 of gestation, and gilt nursing behaviour assessed at d 13 of lactation. Piglets were subject to a back test at d 13 of age, to an open field test and a human approach test at d 20 of age, and growth was monitored to weaning (d 26 of age). Gilts classified as having a fearful behavioural profile had higher cortisol levels than friendly gilts (*p* < 0.05). Human fear level did not affect reproductive performance or the growth of offspring (*p* > 0.05). The offspring of friendly gilts tended to have a more active response to the back test (*p* = 0.09), less freezing response in the open field test (*p* < 0.05), and received human contact more than piglets from fearful gilts (*p* < 0.05). The present study shows that gilt human fear level is linked to their stress levels, which can affect the personality of their piglets.

## 1. Introduction

Gestational and early life experiences influence subsequent behavioural and physical development. Historically, most studies on stressors that affect individuals prior to birth (i.e., pre-natal stress; PNS) have been conducted on non-human primates and rodents, and have demonstrated that offspring from chronically stressed mothers have impaired stress coping ability and locomotor and cognitive development (reviewed in [1]). An example of this in pigs is in relation to maternal behaviour; mothers that are stressed during pregnancy produce daughters that subsequently have poor maternal behaviour (piglet-directed aggression, less motivation to nurse) [2,3]. In most studies to date, PNS has been imposed by carrying out procedures likely to reflect farm practices: from daily physical restraint of the sow [4] to mixing of sows [5,6], to investigate whether maternal stressors, which are reasonably regular occurrences on farm, can also affect the offspring. However other causes of PNS, and their specific effects on the offspring, have not yet been fully investigated in pigs.

Understanding the causes of PNS and how its intensity affects piglet development will help to improve both animal welfare and performance. Unlike in rodents [7], PNS does not appear to affect the weight of piglets at birth [8]. Interestingly, Brajon et al., (2017) [9] found that prenatally stressed piglets showed signs of behavioural inhibition post-weaning (i.e., less environmental exploration and less play behaviour), even though they did not differ pre-weaning. In sheep, PNS, which was mediated by the emotional reactivity of the mother, also altered the behaviour of lambs in both a human approach and a novel object test [10]. Personality is another non-environmental factor that seems to be related to an individual’s physiological and behavioural responses to stress.

One aspect of pig personality is coping style, determined by genetics and early life experience [11]. In pigs, coping style has been most often evaluated in early life using a back test, which assesses the reaction of the pig to restraint (e.g., [12,13,14,15]). Highly responsive pigs, displaying many escape attempts during the test, are considered to have a proactive (or active) coping style, and lowly responsive pigs are considered to have a reactive (or passive) coping style. In stressful situations, proactive pigs are more likely to show an aggressive and less flexible/adaptable behavioural response, and their physiological response (e.g., higher heart rate) is mediated by their sympathetic nervous system. Fear of humans or of novelty are other aspects of pig personality that can affect their productivity (e.g., reproduction, growth rate; [16]) and welfare [17]. Sows that are fearful of humans are more likely to savage their piglets and have stillborn piglets [18], while positive human handling (to promote reduced fear) resulted in shorter farrowing and more rest following farrowing [19]. Human fear level is maintained between parities [18] and although it seems to have a low heritability (e.g., [20,21]), it can be learned by piglets through emotional contagion and social learning [22]. There is a global lack of knowledge on the factors influencing the transmission of human fear from the dam to the piglets. Filling this gap could help improve the human–animal relationship on farm, and therefore promote both animal welfare and productivity.

This study investigated associations between gilts’ fear of humans, gestational stress level, and feeding and maternal behaviour, as well as how these related to aspects of the personality (coping style, fear responses) and growth of their piglets. We hypothesised that sows that are more fearful of humans experience more stress, have poor maternal skills, and that their offspring would also show greater fear of humans, lower growth, and a more proactive coping style.

## 2. Materials and Methods

The experiment was carried out between June 2016 and March 2017 at the Teagasc Pig Development Department, Moorepark, Fermoy, Co., Cork, Ireland. Ethical approval for this study was granted by the Teagasc Animal Ethics Committee (approval no. TAEC120/2016) and the project was authorized by the Health Products Regulatory Authority (project authorization no. AE19132/P051). The experiment was conducted in accordance with Irish legislation (SI no. 543/2012) and the EU Directive 2010/63/EU for animal experimentation.

### 2.1. Animals, Housing and Feeding

Thirty-seven gilts with the same genetic background (Large White x Landrace; Hermitage Genetics, Sion Road, Co., Kilkenny, Ireland) were used in the study. Gilts selected from two breeding batches were artificially inseminated at the onset of standing estrus and again 24 h later using pooled semen (Danish Duroc; Hermitage Genetics, Co., Kilkenny, Ireland). Batches were inseminated at 3-week intervals, with 25 and 12 gilts per batch. During gestation, gilts were managed in a large dynamic group pen which held 120 breeding animals at any one time. The group pen had insulated concrete lying bays and fully slatted floors. Gestating gilts were fed via two electronic sow feeders (ESFs; Schauer Feeding System (Competent 6), Prambachkirchen, Austria) and gilts had ad libitum access to water from single-bite drinkers in the ESFs and from five drinker bowls located around the pen. On d 38 of gestation, gilts were blocked within batch on the basis of body weight (BW; mean ± S.D.; 179.4 ± 9.87 kg) and back-fat depth (BF; 16.9 ± 3.42 mm) and randomly allocated to 1 of 4 dietary treatments until parturition: (1) control (*n* = 8), (2) L-carnitine (0.125 g/d L-carnitine; *n* = 9), (3) sugar beet pulp (40% sugar beet pulp; *n* = 10), and (4) sugar beet pulp plus L-carnitine (40% sugar beet pulp + 0.125 g/d L-carnitine; *n* = 10). This study was a component of a larger study by Rooney et al., (2019) [23], in which the effects of the four different diets were evaluated. For further information on both the dietary treatments and the composition of the experimental diets, please see [23]. Gilts were then moved within their farrowing group to a smaller pen at d 90 of gestation and the pen had the same layout and facilities as the larger group pen. Six days before gilts were due to farrow (d 108 of gestation), they were moved into standard farrowing crates (pen dimensions: 2.5 m × 1.8 m) and farrowing rooms accommodated 7 or 14 animals per room. The health and welfare status of all gilts and their offspring was monitored daily by farm personnel.

Once farrowed, gilts received a standard lactation diet twice daily for the first 6 days of lactation and three times daily thereafter until weaning. Water was provided to gilts from a single-bite drinker in the feed trough and suckling piglets had access to water from a bowl in the farrowing pen. Suckling piglets received creep feed twice daily from d 13 of lactation. The temperature in the farrowing room was maintained at ~24 °C at farrowing and gradually reduced to 21 °C by d 7 of lactation. Artificial lighting was provided from 08:00 h to 16:30 h each day. Where possible, litter size was standardized during the first 48 h after parturition, based on piglet BW, so that there was an average litter size of 13.4 ± 0.40 piglets per gilt. Cross-fostering was only done within gilt treatment and piglets that had been fostered were excluded from further investigations. Therefore, when a gilt is described as the ‘mother’ of a piglet in this study, it refers to the biological mother. Piglets’ teeth were clipped within 24 h postpartum and tails were docked on d 3 postpartum. All piglets received an iron injection on d 5 postpartum and males remained fully intact. Pigs were weaned on d 26 ± 0.1 of lactation.

### 2.2. Categorisation of the Gilts

#### 2.2.1. Human Approach Test

A human approach test (HAT) was performed four times, between d 104 and d 111 of gestation, to assess differences between gilts in their reaction to a human. The HAT was performed when gilts were unrestrained in the pen. The experimenter calmly entered the pen and quietly walked among the gilts for 2–3 min before commencing the test. The test was carried out in a randomised order to control for the order of testing, and the effects on each gilt was tested individually using the scoring system outlined in Table 1 and adapted from [24]. If a gilt voluntarily approached the experimenter, the HAT commenced for that animal and they were assigned 0 for ‘approach’. Otherwise, the experimenter walked slowly towards the gilt from the front. After scoring the gilt’s response to an approach, the experimenter then attempted contact, by reaching out and attempting to touch the gilts neck, and again scoring the reaction. Finally, the type of vocalization, if any, was scored.

#### 2.2.2. Profile Assignment

Gilts were assigned to one of three response profiles each time the HAT was performed: ‘friendly’, ‘fearful’, or ‘unclassified’ (Table 2). Two datasets were then created. The first included only gilts that were categorised as ‘friendly’ or ‘fearful’ in every test (PURE gilts). The second included all gilts. Gilts were categorised as ‘friendly’ or ‘fearful’ if they fit that profile in three out of four HATs, and as ‘unclassified’ if their profile could not be established due to inconsistent scores between tests. In total, 20 gilts were considered PURE: 7 friendly and 13 fearful. When considering all 37 gilts, 15 were classified as friendly, 17 as fearful, and 5 were unclassified.

### 2.3. Gilt Measures

#### 2.3.1. Live-Weight, Back-Fat Depth, and Farrowing Performance

The back-fat depth and live weight of gilts were recorded three times during gestation, at d 38 (blocking), d 90 (move to smaller pen), and d 108 (move to farrowing crates) of gestation, as well as at weaning according to the methods previously described by [23]. The number of piglets born (total, live, and stillborn) was recorded for each litter at birth.

#### 2.3.2. Gestation Feeding Behaviour

Between d 90 and d 108 of gestation (i.e., when in the smaller pen), interactions with the ESF were monitored to determine whether gilt profile influenced feeding behaviour. Each ESF day commenced at 19:00 and concluded at 18:00. Thus, there was an hour during which the ESF was not accessible to the gilts, to enable routine maintenance, etc. The time of each gilts’ first visit after the ESF opened at 19:00 was automatically recorded and downloaded, and from this, the order in which each gilt entered the ESF determined. As there were different numbers of gilts in each batch, the order of entry for each was divided by the total number of gilts in the pen. This value represents the proportion of gilts in the pen that entered the ESF prior to each gilt on each day. The coefficient of variation of the order of entry over all days for each gilt was calculated to provide an estimate of the level of stability of entry order over time. Finally, the total number of ESF visits for each gilt was summed on each day.

#### 2.3.3. Salivary Cortisol

Three saliva samples were collected from gilts and analysed according to the methods previously described by [23]. In brief, samples were collected once every week between d 90 and d 108 of gestation and all samples were collected between 09:00 h and 10:00 h each morning, roughly 9 h after the gilts’ last meal. To obtain the saliva samples, gilts were allowed to chew on a large cotton swab for 30–40 s until it was saturated (Salivette, Sarstedt, Co., Wexford, Ireland). Once collected, the cotton swabs were placed into plastic Eppendorf tubes and centrifuged (400× *g* at room temperature for 10 min), before being stored at −20 °C until analysis. Salivary cortisol concentration was assessed in duplicate using an enzyme linked immunosorbent assay (Salivary Cortisol kit, Salimetrics Europe Ltd., Suffolk, UK). The minimum detectable concentration of cortisol that could be distinguished from 0 was < 0.003 μg/dL. The intra- and inter-assay CVs were 4.5% and 4.2%, respectively.

#### 2.3.4. Nursing Behaviour

To measure the willingness of gilts to nurse their litters, suckling piglets were separated from their mother for 2 h on d 13 post-farrowing. After the 2 h separation period, the gilt was encouraged to stand if she was not already doing so, then her litter of piglets was returned to the farrowing pen. A stopwatch was started when all piglets had been returned to the pen and the time that it took gilts to kneel/lie down and nurse her piglets was recorded. The observation time for each gilt was no longer than 5 min in duration.

### 2.4. Piglet Measures

#### 2.4.1. Growth

The weight and sex of each piglet was recorded at birth, and each piglet was tagged for identification purposes. Thereafter, piglets were individually weighed on d 1, d 6, and d 13 after farrowing, as well at weaning, and the data were used to determine piglet average daily gain (ADG).

#### 2.4.2. Behaviour

**Figure 1:**
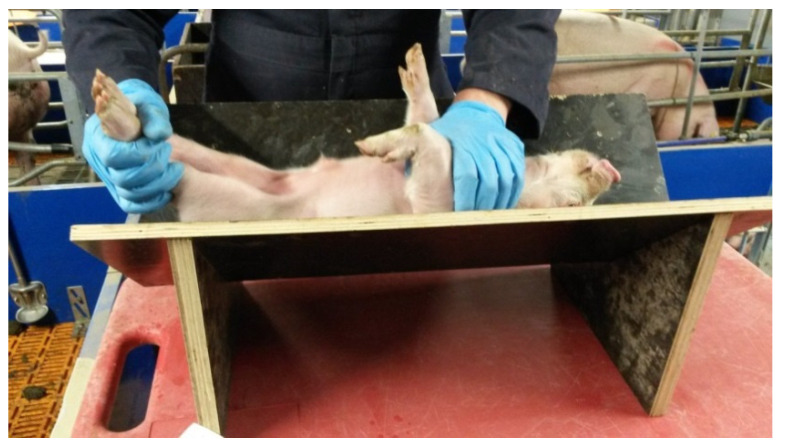
Back-test procedure use on d 13 post-farrowing.

Back Test. On d 13 post-farrowing, each piglet was subjected to a back test as described by [25]. Each piglet was placed on its back on a wooden v-board and restrained for 1 min. The tester held the hind legs with one hand and placed the other gently on the throat (Figure 1). The tester did not change the piglets’ position or move their hands during the 1-min experiment. The piglet was held in this position for 1 min and the number of escape attempts (leg kicks, wriggles) made during this period counted. Each series of wriggles and kicks that was made without pausing was classified as a single escape attempt. The total number of escape attempts was considered the back test score.

Open Field Test. One week after the back-test, on day 20 post farrowing, four piglets were selected for an open field test (OFT) from each of the gilts classified as friendly and fearful (i.e., not including unclassified gilts; *n* = 32 gilts). The piglets selected were those that represented the average BW of the litter and consisted of two males and two females. The back test results were also used in the selection of piglets; piglets were considered a low responder (LR) if the number of escape attempts was less than or equal to 2 and a high responder (HR) if the number was greater than 2 [11,26]. The proportion of high and low responders in the litter was then used to calculate the number of high and low responders to be selected for the OFT (Table 3).

The OFT arena was an empty and disinfected farrowing pen (pen dimensions: 2.5 m × 1.8 m) in a room that was visually and acoustically isolated from the piglets’ home pen. The room that contained the OFT arena was kept at a similar temperature as the other farrowing rooms that housed experimental gilts. Piglets were placed together in a trolley and calmly transferred to the test arena. One at a time, each piglet was then taken out of the trolley and placed in a corner of the pen. As soon the piglet was released, the OFT began. Piglet behaviour (Table 4) was continuously recorded for 3 min by a single observer standing outside the test arena, using a Psion Workabout installed with the software package The Observer^®^ XT (Noldus Information Technology, Wageningen, The Netherlands).

#### 2.4.3. Human Approach Tests

After the OFT test was complete, each piglet was subjected to a HAT in the same test arena. The tester calmly entered the pen, walked to the farthest side, and sat on the floor cross-legged for 1 min. The tester did not move or interact with the piglets during the test, and each piglets’ behaviour was scored based on the following scale: 0: the piglet does not touch the tester during the 1 min test; 1: the piglet touches the tester during the 1 min test. The length of time that it took the piglet to touch the tester was recorded. If a piglet touched the tester during the 1 min test, a forced HAT was performed. The tester initiated contact by slowly moving their hand to the top of the piglet’s head and gently touching the piglet. The piglet’s behaviour in response to being touched was scored based on the following scale: 0:the piglet flees from the contact; 1:the tester touches the piglet but the piglet withdraws after contact; 2: the tester touches the piglet and the piglet withdraws after contact but returns to the tester in less than 10 s; 3: the piglet does not withdraw after contact.

### 2.5. Statistical Analysis

Statistical analyses were performed using SAS 9.4 (SAS Inst. Inc., Cary, CA, USA). The experimental unit for analysis was the gilt. Analyses were carried out on two datasets. The first dataset included gilts that were consistently categorized as friendly (*n* = 7) or fearful (*n* = 13) in all four of the HATs (PURE gilts). The second dataset included all gilts; gilts were categorized as being friendly or fearful if this was how they responded in at least three out of the four HATs. If gilts were friendly and fearful in two each of the tests, they were considered unclassified. This second dataset was analysed firstly to increase the sample size (friendly = 15; unclassified = 5; fearful = 17), and secondly, to determine whether results would be similar if gilts that did not respond completely consistently were included. When using this second dataset, we were able to compare the fearful gilts to all others (friendly and unclassified combined) or to only friendly gilts.

Data distribution and the presence of outliers were initially evaluated by the examination of histograms and normal distribution plots (PROC UNIVARIATE). General linear mixed models (PROC MIXED) were used for most of the analysis. Degrees of freedom were estimated using the Kenwood–Rogers adjustment, and residuals were examined to verify normality and the homogeneity of variances. In cases where repeated measures were used, model fit was determined by choosing models with the minimum finite-sample corrected Akaike information criteria. The Tukey–Kramer adjustment was used for multiple comparisons where least squares means (LS means) were determined (i.e., when we used the second dataset in analysis, and compared all three gilt profiles to each other). Differences were considered statistically significant when alpha was ≤ 0.05, and tendencies were determined when alpha was between 0.05 and 0.1 (inclusive).

All models included the main effects of sow profile (friendly, fearful, and unclassified), as well as the fixed effects of fibre level of the diet (high/low), L-carnitine supplementation (yes/no), and batch. The repeated statement was used where necessary, details of which are provided for specific models below. The gilt was considered the experimental unit in all analysis. Additional terms which were not relevant for all models (e.g., piglet sex, measurement specific covariates, etc.) are detailed in the corresponding section below. To investigate the overall hypothesis that both fearful gilts and their offspring would have responses different to all other gilts, contrast statements were used to investigate differences between fearful gilts and unclassified and friendly combined.

When data did not conform to normality, a transformation was initially attempted (e.g., log transformation for the number of visits per day to the ESF). If unsuccessful, then non-parametric statistic was used. The number of piglets born dead as analysed using the Kruskall–Wallis test with a Dwass, Steel, Critchlow-Fligner procedure to protect against type 1 error.

#### 2.5.1. Sow Measurements

Cortisol measurements from each gilt were averaged over the three sampling days prior to analysis. For analysis of the ESF data, the repeated effect of day was included in the models where relevant.

#### 2.5.2. Piglet Measurements

For the analysis of piglet performance, the sex of the piglet was also included in the analysis. For birthweight, the number of piglets born was also considered a covariate. Piglet birthweight was also included as a covariate for analysis of growth to weaning. Additional factors included in the model for the analysis of the piglet back test were the fixed effects of sex and the person holding the piglets. Birthweight was included as a covariate.

Mixed models were used to analyse the number of low grunts and the duration of standing, walking, and exploration. Aside from the fixed effects included in all models, additional effects included the fixed effect of sex and back-test score.

For analysis of the number of elimination events, screams, and jumps, and the duration of freezing and running, there were multiple 0 values, and as such the data could not be normalised. Thus, data were analysed using the Wilcoxon rank test.

The number of piglets that made voluntary contact from gilts of each profile (friendly vs. fearful) was compared using a Chi-square test. Further analysis could only be carried out on piglets that made contact (*n* = 94). The time it took to touch the researcher was analysed using the same mixed model as for the OFT variables, but log transformed for analysis so that the residuals approached a normal distribution. Once *p*-values were determined, the appropriate model was run using raw data to generate least squares means. The response to the forced human contact test was analysed using the Wilcoxon rank test.

## 3. Results

### 3.1. Sow Measurements

#### 3.1.1. Back-Fat and Weight

Of the PURE gilts, friendly gilts had greater back-fat than fearful (16.8 ± 0.4 vs. 15.3 ± 0.3; *p* = 0.01). However, there was no interaction between recording day and profile, which indicates that friendly gilts simply maintained a back-fat advantage which they had at the beginning of the experiment. When all gilts were included in the analysis, there was no difference in back-fat thickness across profiles (friendly = 16.0 ± 0.4, unclassified = 15.0 ± 0.7, fearful = 15.4 ± 0.3). Live-weight did not differ across the profiles, whether only PURE or all gilts were included in the analysis.

#### 3.1.2. Cortisol

**Figure 2:**
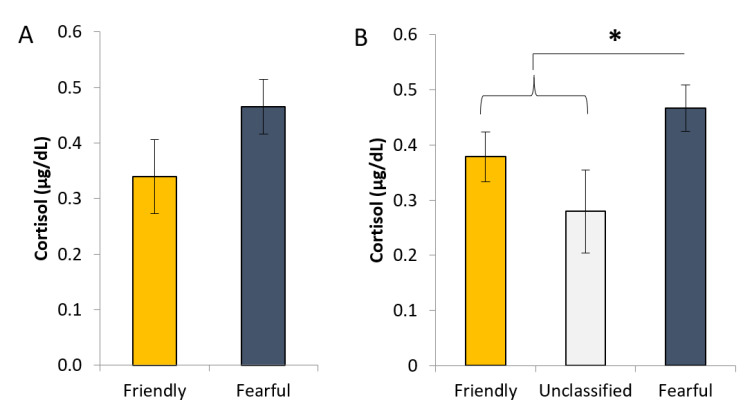
Salivary cortisol levels of gilts categorised to different behavioural profiles which were (**A**) consistently friendly or fearful in four human approach tests (HAT) and (**B**) were either friendly or fearful in three out of four tests, or considered unclassified (two friendly and two fearful outcomes). * indicates a difference at *p* < 0.05 for the contrast between fearful gilts and friendly and unclassifed combined.

There was no difference in cortisol level between the PURE friendly or fearful gilts (*p* = 0.15; Figure 2A). However, when all gilts were included in the analysis, there tended to be an effect of gilt profile (*p* = 0.1), with fearful gilts having higher cortisol levels than the other categories (*p* < 0.05; Figure 2B).

#### 3.1.3. Feeding Behaviour

For the PURE gilts, there was no effect of profile on the order in which the gilts entered the ESF (fearful = 54 ± 5% vs. friendly = 56 ± 6%; % represents the proportion gilts in the group that entered the ESF prior; *p* = 0.77). When all gilts were included in the analysis, the effect of profile was significant (*p* < 0.01). However, this was driven by the unclassified gilts, which on average entered the ESF much later (75 ± 8% entered prior to them) than either the friendly (45 ± 4%; *p* < 0.01) or fearful (56 ± 4%; *p* = 0.07). There was no difference between the friendly or fearful (*p* = 0.13).

**Figure 3:**
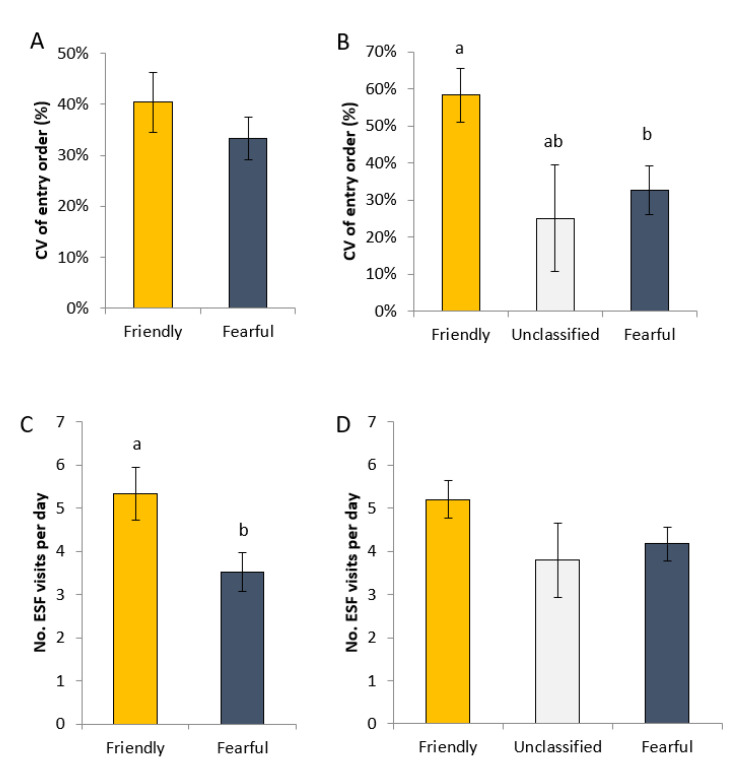
The coefficient of variation of the order in which gilts entered the ESF which were (**A**) consistently friendly or fearful in four human approach tests (PURE gilts) and (**B**) the same measure for gilts that were either friendly or fearful in at least three out four tests or considered unclassified (two friendly and two fearful outcomes). The number of visits per day to the ESF for (**C**) PURE gilts and (**D**) all gilts. ^a,b^ indicates a difference at *p* < 0.05, when Tukey’s adjustment was applied post-hoc.

For the PURE gilts, there was no effect of profile on the variation in the order in which the gilts entered the ESF over the experimental period (*p* = 0.34; Figure 3A). When all gilts were included in the analysis, however, the effect of profile became significant (*p* < 0.05), with friendly gilts displaying more variation than fearful gilts (*p* < 0.05; Figure 3B).

Of the PURE gilts, friendly gilts entered the ESF more times per day than the fearful (*p* < 0.01; Figure 3C), and this pattern was somewhat replicated when all gilts were included in the analysis (Figure 3D), as there tended to be an effect of profile (*p* = 0.1). However, in this scenario there was no significant difference between friendly and fearful gilts (*p* = 0.17).

#### 3.1.4. Reproductive Performance

There were no effects of gilt profile on reproductive performance (Table 5).

#### 3.1.5. Nursing Behaviour

**Figure 4:**
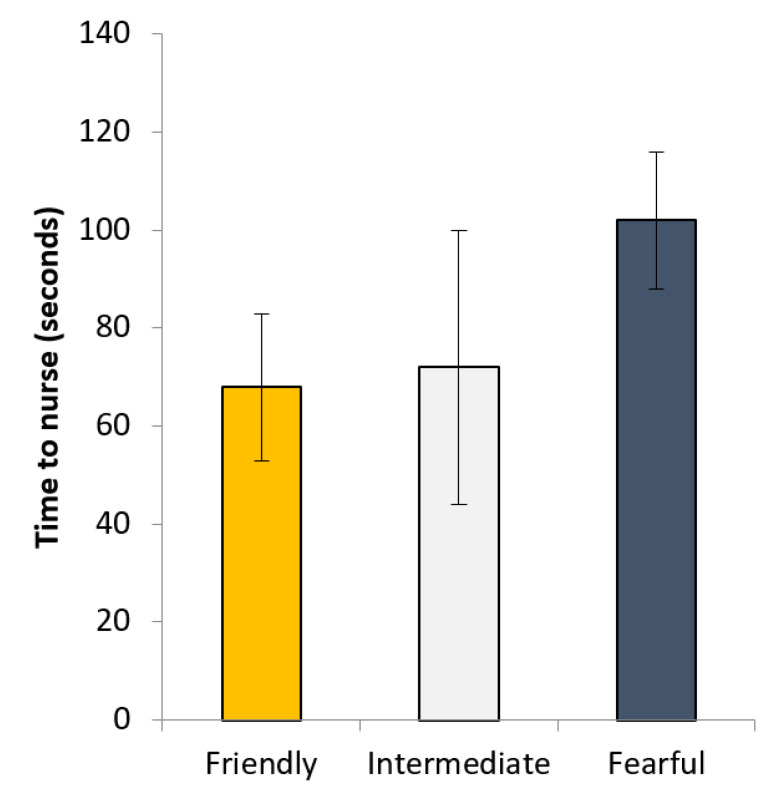
Time taken for sows to lie down and nurse their piglets after piglets had been removed from the pen for 2 h.

There was no difference in time to nurse piglets after a 2 h separation, when considering only PURE gilts. However, when including all gilts in the analysis, although there was no overall difference in time to nurse, fearful gilts tended to take longer to nurse than all others (*p* = 0.1; Figure 4).

### 3.2. Piglet Measurements

#### 3.2.1. Piglet Performance

**Figure 5:**
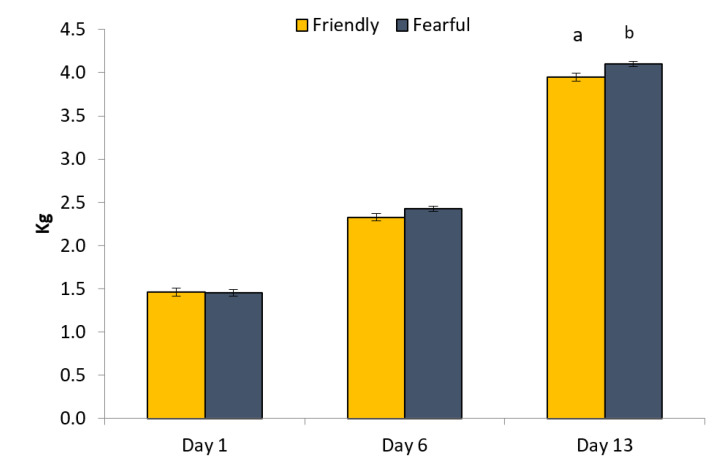
Live-weight of piglets from sows that were categorised as friendly or fearful in four human animal approach tests. ^a, b^ indicates a difference at *p* < 0.1 > 0.05.

There was no effect of gilt profile on piglet birthweight, whether only PURE or all gilts were included. Across all gilts, piglets weighed approximately 1.32 ± 0.34 kg (mean ± std. dev.) at birth. When all piglets were included in the analysis, there was no effect of gilt profile on the piglets’ growth to weaning or weaning weight. However, when only piglets from the PURE gilts were included, piglets from fearful gilts tended to be heavier than the friendly (*p* = 0.05), and there also tended to be an interaction between profile type and weighing data (*p* = 0.07). Indeed, on day 13 after birth, piglets from the fearful gilts tended to be heavier than those from the friendly ones (*p* = 0.06; Figure 5). However, weaning weight did not differ for piglets from sows of divergent profiles. All piglets were weaned at approximately 7.02 ± 1.53 kg.

#### 3.2.2. Back-Test

When piglets from all gilts were included in the analysis, there was no effect of sow profile on back-test scores. However, when only considering PURE gilts, piglets from friendly gilts tended to have a higher back-test score than the fearful (3.47 ± 0.21 vs. 3.01 ± 0.15; *p* = 0.09).

#### 3.2.3. Open-Field Test

There was no effect of gilt profile, whether including all gilts or only the PURE ones, on the duration of standing, walking, exploring, or running. However, when all gilts were included, piglets from friendly gilts spent less time in a freeze position (0 (0–3.53) seconds, median (interquartile range)) than fearful ones (2.61 (0–9.42) seconds; *p* = 0.01). Likewise, when only considering PURE gilts, piglets from friendly gilts tended to spend less time frozen (0 (0–4.11) seconds) than piglets from fearful gilts (2.51 (0–8.89) seconds; *p* = 0.06). There were no differences in the incidence of low- or high-pitched vocalisations, eliminations, or jumping.

#### 3.2.4. Human Approach Tests

At least one piglet from every sow voluntarily made contact with the tester. When all gilts were included, piglets from friendly gilts tended to be more likely to make contact with the tester than piglets from fearful gilts (*n* = 127 piglets; 81.4% vs. 67.6%; *p* = 0.1). However, this was not the case when only PURE gilts were considered, even though the proportions were similar (*n* = 79 piglets; 81.5% vs. 69.2%; *p* = 0.29).

When data from all gilts were analysed, piglets from friendly gilts took less time numerically to touch the tester than the fearful (17.1 ± 2.9 vs. 19.3 ± 2.7 s; *p* = 0.15), but this was not significant. When data from only PURE gilts were analysed, there was a tendency for piglets from friendly gilts to take less time, even given the smaller sample size (friendly = 12.8 ± 2.9 vs. fearful = 16.8 ± 2.3 s; *p* = 0.08).

For the forced human contact test, scores of piglets from friendly gilts were higher (i.e., indicative of less fear) than those from fearful gilts (2 (1–3) vs. 1.5 (0–3); *p* = 0.03). When only considering PURE gilts, the pattern was similar (friendly = 2 (2–3) vs. fearful = 1.5 (0–3); *p* = 0.04).

## 4. Discussion

In this study, we investigated the effects of gestating gilts’ reaction to a human and the associated stress level on their feeding and maternal behaviour and on the personality (coping style, human fear) and growth of their piglets. Gilts classified as having a fearful response (determined by HAT scores) had higher basal cortisol levels during late gestation compared with friendly gilts. Contrary to expectations, human fear level did not affect the growth of offspring from birth to weaning. However, a strong association between prenatal stress and the personality or coping behaviour of gilt offspring was observed in the behaviour tests applied to piglets, whereby behaviour responses that indicate an increased level of fearfulness were observed in offspring of fearful gilts.

Fear of humans is often considered an important indicator of farm animal welfare, as it is associated with physiological stress and can influence maternal performance [19,29]. Cortisol concentration in saliva is often used to assess the level of physiological stress the animal is experiencing, as it correlates well with circulating levels in blood [30,31]. As such, salivary cortisol concentration has often been used as a measurement to give insight into the effects of PNS and negative handling, and on types of human–animal interaction. Jarvis et al., (2006) [2] demonstrated that sows exposed to an environmental stressor during gestation (stimulated PNS via social mixing) had increased salivary cortisol levels; while several studies have shown that animals that experienced unpleasant handling have higher cortisol concentrations [32,33,34]. In the present study, the higher basal cortisol levels of gilts that were classified as being fearful rather than friendly during late gestation (between day 90 and day 108) suggests that differences in HAT scores may be reflected in physiological responses. This finding agrees with those of a recent study, whereby pigs that were more fearful (based on results from a novel object test) had significantly higher levels of cortisol at slaughter [35]. There are potential limits when interpreting cortisol data, as the average concentrations of cortisol in saliva are influenced by several factors such as age (concentrations decrease with age), sex (higher concentrations in males than in females; [36]), and time of the day (levels peak in the early morning hours and are lowest in the evening and at night) [37]. However, we controlled for these factors by collecting saliva samples from all gilts in each group on the same day and within an hour (usually within 15 min) for all subjects.

In addition to the greater variation in entry order, friendly gilts also had a greater number of visits to the ESF per day than fearful gilts. A greater number of entries into the feeding stations could mean that friendly gilts were more ‘optimistic’ that they would be fed each time they entered the ESF. This hypothesis can be compared to judgement bias tests, an alternative measure for evaluating psychological welfare in animals [38]. The theory behind this test suggests that an animal will evaluate a particular stimulus as predicting either a positive or negative outcome, depending on the animal’s affective state [38,39,40]. As such, friendly gilts could have associated each visit to the ESF as having a positive outcome (i.e., receiving feed upon entry to the ESF). If the feeding behaviour of sows and gilts that were housed in large loose housing and fed via ESF stations was studied consistently, the data could then be harnessed to assist with the identification of animals needing specific attention, similar to the use of automatic milking robots in dairy systems [41], or utilised as a non-invasive measure of optimism/pessimism within a breeding herd.

Although differences in HAT scores were reflected in cortisol levels during pregnancy, reproductive performance (numbers total born, born alive, and stillborn) did not differ between gilts with different behaviour profiles. Indeed, average cortisol levels across gilts were lower than those recorded in gestating gilts in the same research facility [42], and lower than the levels of the larger pool of gilts from which the current study animals were selected [23]. Thus, there is no indication that fearful gilts were excessively stressed relative to the other profiles, rather just that they had higher basal cortisol levels, which is possibly why foetal growth was not affected. Where foetal growth was previously shown to be affected by increased maternal cortisol levels, it was generally where substances were administered to initiate a state of physiological stress artificially; oral administration of hydrocortisone acetate to sows during early and late gestation resulted in decreased piglet birthweights [43], and the birthweight of piglets born to sows treated with injections of adrenocorticotrophic hormone during the last week of pregnancy was lower than piglets from non-treated sows [44]. Our findings are consistent with previous work on pigs (see review by [45]), whereby stressful conditions during gestation had no effect on the weight of sow progeny at birth. The aforementioned results suggest that the growth of offspring in utero can be influenced by artificially increased maternal cortisol levels during gestation (i.e., glucocorticoid models) but that the experience of stressful situations (e.g., those imposed by management) by the pregnant sow have little or no effect on foetal growth. However, it must be noted that the sample size used in the present study was likely too small to detect significant differences in the reproductive performance of gilts.

We used a simple test of nursing behaviour as an indicator of maternal behaviour. Our original hypothesis that gilts that are more fearful of humans have lower maternal skills was not entirely supported, however, as there was little difference in the response to piglets after a separation. Nevertheless, the small sample size in the current study may have precluded the ability to detect a difference, as the numerical pattern was indicative of a longer latency to lie in fearful gilts. There is other evidence in the literature that fearful behaviour during gestation translates to poor maternal ability; Marchant Forde et al., (2002) [18] reported that a fearful behavioural profile during gestation was associated with increased savaging in gilts and Rutherford et al., (2014) [3] observed more abnormal maternal behaviour (e.g., less time spent lying laterally and increased restlessness) in gestating sows that experienced the social stress of mixing, relative to those in stable groups. Janczak et al., (2003) [29] also found that sows showing less fear of humans had more adaptive maternal behaviour and concluded that fear of humans is negatively associated with maternal ability. Future work could aim to validate our simple test, taking into account sample size and precise profiling of sows.

The coping style of an individual pig is a reflection of its preferred strategy for reacting to stressors [46]. In the present study, piglets from friendly gilts tended to have a higher back-test score (i.e., they made more escape attempts during the back test and thus were classified as high-responsive piglets) than piglets from fearful gilts. Thus, they adopted a more active coping style in response to the stress of being restrained than piglets from fearful gilts, which displayed a more passive coping style. This result suggests that the coping style of offspring in response to stress or fear might be somewhat influenced by the fearfulness, or stress levels during gestation, of the dam. However, to obtain more robust data on this relationship, more behaviour testing incorporating a wider range of test types that contribute to personality profiling for the mother is advised for future studies [14]. It is not possible to elucidate from our data any causal relationship between maternal fear, maternal cortisol levels, and piglet responses.

Nevertheless, we did incorporate into the present study two other behaviour tests for piglets in addition to the back-test. The HAT and the open field/novel environment test allow for the assessment of fear of both humans and novelty and are complementary to the back test. For example, Kooij et al. (2002) [47] demonstrated a correlation between the back-test score of pre-weaned piglets and the behaviour of piglets in a HAT that was conducted after weaning (5–7 weeks of age). In the aforementioned study, proactive piglets were more likely to approach a human faster than a reactive pig during the HAT. Likewise, piglets from friendly gilts in the current study were considered to have a proactive coping style according to the back test, and also tended to be more likely to make contact with the tester during the HAT and be more likely to permit the human experimenter to make contact with them. Therefore, our findings suggest a relationship between the level of fear expressed by gilts, and their offspring, towards humans.

There also appeared to be a clear relationship between the response to the back test and the behaviour of piglets in another stressful situation, the open field test; piglets from friendly gilts, which were generally proactive, spent less time in the freeze position during the open field test than piglets from fearful gilts (generally reactive). Again, this is consistent with the results of former studies [12,15,26]. For example, Zebunke et al., (2017) [15] demonstrated that piglets that were classified as high responders showed earlier contact with an unknown human in a human approach test. Furthermore, the high responders exhibited earlier and more frequent but shorter locomotion and standing episodes, longer contact with a novel object, and shorter latency until the first escape attempt in an open field test. Our results are also in agreement with the description of Koolhaas et al., (1999) [48] that proactive animals engage in an active response, also known as ‘fight-flight’, to challenging situations whereas reactive animals engage in a conservation-withdrawal response, also known as ‘freeze’.

It is beyond the scope of this study to consider whether the responses of the piglets to the various behaviour tests and their associations with maternal profile were due to genetic, epi-genetic, or environmental effects (e.g., exposure to cortisol, learning from the mother). However, it is possible that it was a combination of all three; as well as the documented effects of exposure to cortisol (listed above), there is evidence that personality type in animals is somewhat heritable. For instance, Dochtermann et al., (2015) [49] found that up to 52% of variation in animal personality could be due to additive genetic variation. Our study does indicate however that associations are present and as such, it is a useful addition to the literature from which future hypotheses can be developed.

Finally, our original hypothesis that PNS during pregnancy may be negatively associated with the subsequent growth of offspring was not supported by our results as the overall growth rate of piglets from birth to weaning was unaffected by gilt behavioural profile. Piglets from gilts classified as consistently fearful (i.e., PURE fearful gilts) tended to be heavier than piglets from consistently friendly gilts at 13 days of age, but this did not translate into heavier piglets at weaning.

## 5. Conclusions

Given the pressure for efficiency in pig farming from both an economic and overall sustainability point of view, transmission of both fearful behaviours and poor maternal skills should be avoided. Our results demonstrate a clear relationship between gilt behaviour and physiology during pregnancy and maternal behaviour during lactation. In turn, the offspring of mothers fearful of humans had a behaviour profile indicating that they were also fearful. We found that the patterns of the results were very similar whether we included gilts which were completely or only partially consistent in their response to a human approach test, which is useful information for the planning of future research where sample sizes may be limited. Further work could build on our results and attempt to validate quick-to-use and easily applied methods to identify pigs that may be more fearful and need special attention/care (e.g., data automatically collected by the ESF). This is particularly the case for replacement gilts, as fear of humans could result in impaired welfare and performance not only for themselves, but also for their offspring.

## Figures and Tables

**Table 1:** Scoring system used to access gilt response to a human experimenter. HAT scoring system adapted from [24].

Score	Approach	Contact	Vocalization
0	Gilt approaches the tester.	Gilt does not move or change behaviour.	Gilt does not make any noise upon attempt to contact.
1	Gilt ignores the tester.	Gilt changes her position calmly: she stands or sits if she was lying for instance.	Gilt grunts gently. The sound is deep. It does not last a long time (<3 s).
2	Gilt moves away when the tester approaches.	Gilt flees the tester passively, without aggressive behaviour, when touched.	Gilt grunts loudly, persistently, for the duration of the attempt at contact. The sound is more high-pitched.
3		Gilt reacts aggressively, shakes her head, or even tries to bite the tester.	

**Table 2:** Behaviour profiles assigned to gilts according to scores received during four human approach tests performed between d 104 and d 111 of gestation.

Profile	Definition	Approach	Contact	Vocalisation
Friendly	Gilt is calm, not aggressive, not fearful.	0 or 1	0	0 or 1
Fearful	Gilt avoids contact, with or without aggression.	2	1, 2, or 3	2
Unclassified	The gilt profile has not been established because her behaviour does not conform consistently to either profile.	Any other combination of scores.		

**Table 3:** The number of piglets which were high or low responders selected per litter for an open field test, relative to the proportion of high and low responders in the entire litter ^1^

% HR in Litter	High Responder	Low Responder	% LR in Litter
0–12.5	0	4	87.5–100
12.5–37.5	1	3	62.5–87.5
37.5–62.5	2	2	37.5–62.5
62.5–87.5	3	1	12.5–37.5
87.5–100	4	0	0–12.5

^1^ Piglets were back-tested and weighed on d 13 post-farrowing and considered a high responder if the number of escape attempts was > 2 and a low responder if number was ≤ 2; HR, high responder; LR, low responder.

**Table 4:** Ethogram of behaviours recorded by continuous observation during the open field test. Adapted from [27,28].

Behaviour	Description
Behaviour states (duration, mutually exclusive)	
Stand	Stationary with all four feet on the floor.
Lie	Stationary with body in contact with the floor.
Walk	Moving slowly in a forward or backward direction or turning around at the same location, with head up.
Explore	Investigating the floor, walls, or fittings of the arena by sniffing, nosing, licking, rubbing, or rooting it with the rooting disc. Rooting disc is either in contact or very close to the surfaces being explored.
Freeze	Duration and frequency of complete immobility and absence of vocalization.
Run	Moving rapidly around the arena in an attempt to escape.
Point events (incidence recorded)	
Low-pitched vocalization	Short or long grunts.
High-pitched vocalization	Screams and squeals.
Elimination	Defecating or urinating.
Jump	Jumping in air or against a wall of the arena.

**Table 5:** Reproductive performance of sows considered friendly, unclassified, or fearful in four human approach tests. Unless otherwise indicated, data are presented as least squares means ± standard errors.

Reproductive Performance	Friendly	Unclassified	Fearful	*p*-Value
Pure gilts				
Gestation duration (d)	115.5 ± 0.6		115.6 ± 0.4	0.97
Total born	14.31 ± 0.67		15.17 ± 0.48	0.31
Born alive	13.38 ± 0.76		14.53 ± 0.54	0.23
Still born	1.0 ± 0.8 ^1^		0.5 ± 0.5 ^1^	0.15
All gilts				
Gestation duration (d)	115.5 ± 0.3	115.6 ± 0.6	115.5 ± 0.3	0.99
Total born	14.12 ± 0.61	14.10 ± 1.10	14.41 ± 0.55	0.93
Born alive	13.35 ± 0.64	12.35 ± 1.14	13.94 ± 0.57	0.45
Still born	0.8 ± 0.9 ^1^	1.8 ± 2.8 ^1^	0.5 ± 0.5 ^1^	0.53

^1^ Data presented as raw means ± standard deviation.

## Data Availability

Not applicable.
